# *Galba truncatula*: Distribution, Presence in Fountains and Identification of Factors Related to Its Occurrence in Bulgaria

**DOI:** 10.3390/ani16020226

**Published:** 2026-01-12

**Authors:** Katya Georgieva, Boyko Neov

**Affiliations:** Institute of Biodiversity and Ecosystem Research, Bulgarian Academy of Sciences, 1113 Sofia, Bulgaria; boikoneov@gmail.com

**Keywords:** *Galba truncatula*, distribution, habitats, environmental factors, logistic regression, Bulgaria

## Abstract

*Galba truncatula* acts as an intermediate host for several parasitic flukes of veterinary importance, but a targeted study on its spatial presence as well as the impact of environmental factors in Southeastern Europe has not been conducted. During the summer months of 2017 and 2018, a survey of 191 water bodies in 14 districts in Central, Southern and Western Bulgaria was conducted, with a focus on animal drinking fountains. Each site was assessed for snail presence and characterized by altitude, temperature, precipitation, shade and type of water body. Logistic regression modeling was used to identify the important factors related to the occurrence of snail species. The frequency of habitats found was 29.3%, with no differences observed between the studied districts (*p* > 0.05). Snails were present across a wide range of altitudes (78–1926 m), annual mean temperature (7.8–14.0 °C) and annual mean precipitation (523–796 mm). The high habitat frequencies were recorded in streams (60.0%) and on the banks on small rivers (50.0%). The presence of snails in the two studied types of fountains (without or with a concrete platform) was 24.1% and 17.2%, respectively, with no significant difference between them (*p* > 0.05). Regression analysis revealed temperature, shade, and type of water body as factors that could significantly influence the spatial presence of *G. truncatula*. The findings demonstrate the ecological adaptability of *G. truncatula* and highlight its presence in habitats with high potential for contact with domestic and wild ruminants. This information fills a regional knowledge gap and can support risk assessment and control measures for fluke-borne diseases in livestock and wildlife.

## Simple Summary

The small freshwater snail *Galba truncatula* spreads parasites that harm livestock and wildlife, but data on its distribution and habitat preferences in Southeastern Europe are scarce. This study covers 191 water points in Bulgaria, focusing on animal-watering fountains, to map where the snail lives and to explore how factors as altitude, temperature, precipitation, shade and type of water body affect its occurrence. A logistic regression model was constructed to assess the importance of environmental factors in the occurrence of snail species. *G. truncatula* was found at 56 locations (29.3%), including in regions where it had never been recorded before. Preferred habitats were streams (60%) and banks on small rivers (50%), but presence in artificial fountains was up to 24%. Temperature, shade and type of water body were identified as important factors, determining the occurrence of *G. truncatula*. The results show that *G. truncatula* can adapt to many habitats, including those frequented by domestic animals, which increases the risk of parasite transmission. Mapping its spread helps predict and manage diseases such as liver and rumen fluke infections in livestock, supporting better animal health, reduced economic losses for farmers, and improved protection of wildlife in Bulgaria and the wider region.

## 1. Introduction

The lymnaeid snail *Galba truncatula* is found in all countries of Europe, but it also occurs in freshwater ecosystems of Africa, Asia, North and South America [1]. The scientific interest in this freshwater snail is mainly due to its role in the transmission of trematodes of health and economic importance as *Fasciola hepatica* (liver fluke) [2,3] and *Calicophoron daubneyi* (rumen fluke) [4]. Human fasciolosis is rarely reported in European countries [5,6], but is a serious problem in other areas of the world [7]. For the livestock industry, both trematodes cause diseases and economic losses, regardless of the preventive and control measures applied [8]. Fasciolosis also affects wild ungulates and causes damage to game farming [9,10,11]. In Central Europe, wild ruminants are affected by the invasive trematode *Fascioloides magna* (American liver fluke), whose vector is also *G. truncatula* [12,13,14].

*G. truncatula* is an intermediate host for trematodes such as *Haplometra cylindracea* [15], *Tylodelphis* sp. [16], *Notocotylus* sp., *Plagiorchis* sp. and *Opisthioglyphe* sp. [17], whose definitive hosts are frogs, fishes and birds. These parasites are important for maintaining the stability of freshwater ecosystems and regional biodiversity, making this relationship another focus of scientific interest regarding this snail species.

*G. truncatula* occurs in the same biogeographic regions as humans, domestic and wild animals. Its presence in aquatic habitats is determined by environmental factors, the exact values of which vary widely. All available information about the characteristics of the natural environment of *G. truncatula* has been recently summarized by Smith et al. [18]. Data on this topic have been obtained mainly from studies in Western European countries. However, insights gained from these specific regions cannot be directly applied to areas with different climatic and geographical characteristics. To develop effective prevention and control measures, as well as to predict future changes, it is essential to gather specific ecological information from the unexplored biogeographic areas within the snail’s extensive range.

Bulgaria is a Southeast European country and occupies the eastern part of the Balkan Peninsula, located between 41 and 44 degrees North latitude and 22 and 28 degrees East longitude, in the southern part of the temperate climate zone. The mountainous and semi-mountainous regions occupy 42.5% of the country’s territory, providing good conditions for the survival of *G. truncatula*. Livestock farming is a major industry in Bulgaria, employing about 15% of the country’s agricultural workforce [19]. Animal husbandry is primary pasture-based. Trematode infestations are a significant concern, with data showing distinct patterns among species. In sheep, the prevalence is dominated by *Paramphistomum* sp. (58.92%) and *F. hepatica* (17.26%). Goats show a different pattern, with infestation rates of 33.33% for *Paramphistomum* sp. and 20.00% for *F. hepatica* [20]. For cattle, the most recent data—dating from 1980—indicate *Paramphistomum* sp. infestation rates ranging from 31.9% to 52.2% across different regions of the country. Furthermore, a parasitological survey in the hunting farms detected the presence of *Paramphistomum* sp. in *Cervus elaphus*, *Dama dama* and *Ovis orientalis* [21]. According to Sey and Vishnykov [22], paramphistomid specimens collected in localities in Bulgaria belong to a single species: *Calicophoron daubneyi*. The intermediate host, *G. truncatula*, plays a key role in the transmission of these trematodes. However, to our knowledge, studies on its occurrence and habitat characteristics have not been conducted in Bulgaria for over 40 years. Such data are also lacking for the broader Southeastern Europe region.

The objectives of this study were: (1) to detect and map the habitats of *G. truncatula* in different biogeographic regions of Bulgaria—the focus of study was on the search for *G. truncatula* in rarely studied water sources, namely artificially created fountains for animal watering, which are associated with local livestock practices and are common throughout the country; and (2) to identify the environmental factors with a role in the occurrence of the snails in the study area.

## 2. Materials and Methods

### 2.1. Study Area

**Figure 1:**
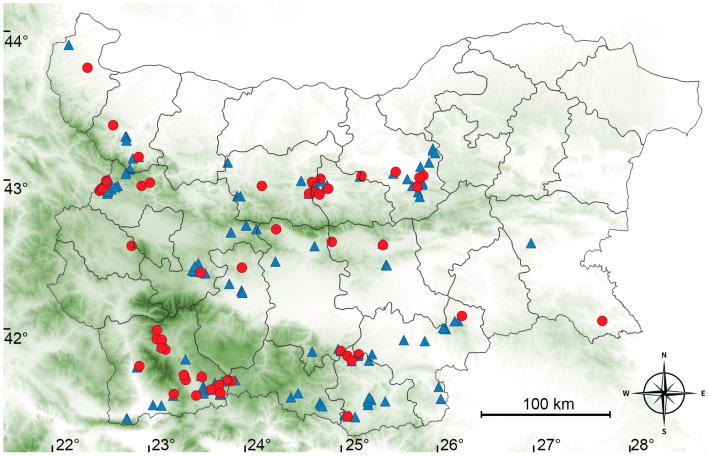
Map of Bulgaria with the outlines of all 28 districts, 14 of which were visited in July–August 2017 and July–August 2018. Geographic longitude and latitude are given in degrees “East longitude” and “North latitude”, respectively. Localities where samples of *G. truncatula* were found and collected are marked in red.

The search for *G. truncatula* habitats was conducted on the territory of 14 districts (an administrative unit in Bulgaria that includes several municipalities) located in Central, Southern and Western Bulgaria (Figure 1, Table 1). An area of 66,255.6 km^2^ was surveyed, which is 59.7% of the country’s area. The relief is varied, with an altitude between 0 and 2925 m. The climate is continental modified by mountainous conditions such as lower temperatures and increased precipitation with elevation.

### 2.2. Sampling Location

A total of 191 water points were surveyed once during the authors’ field research in July–August 2017 or July–August 2018 and the fauna at each site was examined for the presence of *G. truncatula* (Figure 1). These months were chosen to maximize the detection of snails in their habitats. This period precedes the autumn movement of herds to high- mountain pastures, a traditional practice for many regions of the country, caused by the appearance of the so-called “fluke fields”.

The majority of the country’s livestock is concentrated in the studied regions [23]. The majority of them are classified as difficult to access due to their natural characteristics [24]. The investigated sites were randomly selected based on their proximity to urban and rural areas, livestock routes, pastures with year-round access for domestic animals, or in forested areas. Snails were searched in potential freshwater habitats, which were not connected or fed by the same water source. The presence of specific algae suitable as snail food at a given site was used as a criterion for determining its suitability for the study. The most common water bodies were artificial roadside fountains for livestock watering. Information about the studied sites (sampling date, GPS coordinates, altitude, shade cover, water body type and photographs) was recorded on site (Supplementary Materials, Additional file: Table S1). Snails were searched visually in delineated 4–5 quadrants of 1 m^2^ at each visited site, for a standardized period of 15 min, by two people. The map of the study area with the locations was created using the Geographic Information System (GIS) 3.44.1 “Solothurn” software [25].

### 2.3. Collection of G. truncatula Snails

**Figure 2:**
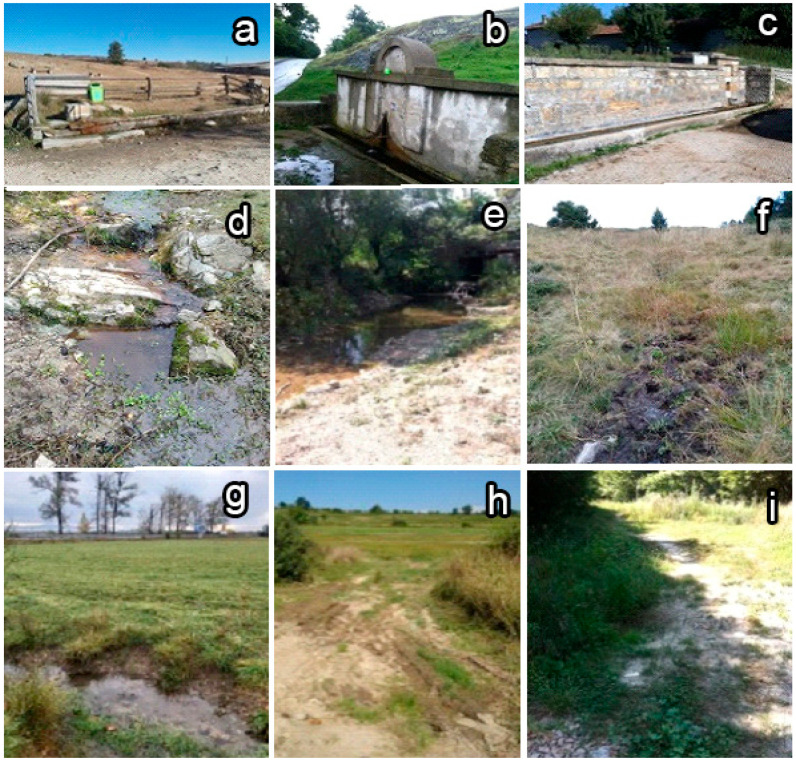
Types of water bodies surveyed for the presence of *G. truncatula*. (**a**)—Type A—Fountain for animal watering with/without trough(s); (**b**,**c**)—Type B—Fountain for animal watering with trough(s), surrounded by a concrete platform; (**d**)—Type C—Stream (up to 1 m in width); (**e**)—Type D—Small river; (**f**–**i**)—Type E—Other; (**f**)—Spring, (**g**)—Irrigation ditch; (**h**)—Drying up puddle; (**i**)—Dried-up puddle.

If *G. truncatula* was present, only adult snails (>4 mm shell height) were collected. In the fountains, which were distinguished by the presence of a concrete platform (Figure 2b,c), snails were collected from the water-soaked surfaces surrounding them or directly from the trough(s). A maximum of 10 specimens per site were collected using tweezers. They were placed in plastic containers with water from the source (when available) and a portion of the aquatic vegetation present at the site. Containers were stored in a refrigerator bag during transport to the laboratory. All collected specimens were provisionally identified in situ by shell morphology. Immediately upon completion of each field trip, the collected material was reexamined under a stereomicroscope to confirm the correct identification, based on morphological characteristics based on Georgiev [26] (p. 155) and our experience. Any specimens that were not unambiguously identified as *G. truncatula* were excluded from subsequent analysis.

### 2.4. Environmental Data and Analyzed Parameters

The altitude of each sampled site was recorded at the time of visit using a handheld global positioning system receiver (GPS Status 11.4.316, http://mobiwia.com/gpsstatus (accessed on 16 August 2018)). For each administrative district studied, mean annual temperature and precipitation data for 2017 and 2018 were sourced from meteorological stations in Bulgaria (data available from the National Institute of Meteorology and Hydrology (https://bulletins.cfd.meteo.bg (accessed on 5 September 2023)). These continuous variables (altitude, temperature, precipitation) were then categorized into three intervals to facilitate analysis and mitigate the influence of extreme values. The categorization was as follows: for altitude (<500 m/501–1000 m/> 1000 m); for temperature (7.8 °C, 9.8 °C/12.2–12.8 °C/13.0–14.0 °C); for precipitation (523 mm, 532 mm/603–686 mm/702–796 mm). Additionally, shade cover at each site was assessed visually and classified into three categories: absent, partial, or present. The studied water bodies were grouped as follows: A—fountains for animal watering with/without trough(s); B—fountains for animal watering with trough(s), surrounded by a concrete platform; C—streams (up to 1 m in width); D—small rivers; E—others (springs, irrigation ditches; puddles) (see Figure 2).

The data collected were used to: (1) determine the frequency of snail habitats (the ratio between the number of positive sites for *G. truncatula* and the number of sites visited) in each: (a) study district; (b) category of temperature, altitude, precipitation, shade cover and type of water body; (2) constructing a logistic regression model.

### 2.5. Statistical Analysis

Statistical analyses were performed using R 4.4.1 software [27]. Due to the violation of requirements for conducting a parametric test, Kruskal–Wallis ANOVA with Dunn post hoc analysis was used to test for differences in *G. truncatula* habitat presence among the 14 districts studied and between categories of the other independent variables. A Mann–Whitney test was applied to compare temperature/precipitation values in 14 districts for the two study years. During the preliminary analysis, the relationship between altitude, temperature and precipitation in the study areas was analyzed using Pearson’s correlation coefficient (Table 2).

Multivariable binary logistic regression was used to identify the environmental variables associated with the occurrence of *G. truncatula* snails. Snail presence/absence at each of the 191 sampling sites was defined as the binary dependent variable (0 = absence, 1 = presence). Five independent variables were included: altitude, mean annual temperature, mean annual precipitation, shade presence, and type of water body. Collinearity between these variables was further assessed by calculating variance inflation factors (VIFs). To build the optimal model, a stepwise mixed selection procedure was employed, starting with a full model containing all independent variables and their possible interactions. Candidate models were then compared, and model fit was evaluated using the Akaike Information Criterion (AIC). The model with the lowest AIC value was retained for final interpretation.

## 3. Results

### 3.1. Habitat Frequency of G. truncatula

The distribution of sampling sites and the identified *G. truncatula* habitats are presented in Figure 1, and Table 1 and Table S1. Snails were found in 56 of the 191 surveyed sites, corresponding to a habitat frequency of 29.3%. There was no significant difference in habitat frequency among the 14 studied districts (χ^2^ = 7.21, df = 13, *p* > 0.05).

The frequency of *G. truncatula* habitats according to altitude, temperature, precipitation, shade and type of water body is shown in Table 3. The occurrence of habitats differed significantly only within the groups of the variable “Type of water body” (χ^2^ = 15.30, df = 4, *p* < 0.01). Dunn’s test revealed differences between fountains type A and streams (type C), and between fountains type B and streams (type C).

### 3.2. Environmental Factors and Occurrence of G. truncatula

#### 3.2.1. Characteristics of the Altitude, Temperature and Precipitation in the Study Area

**Figure 3:**
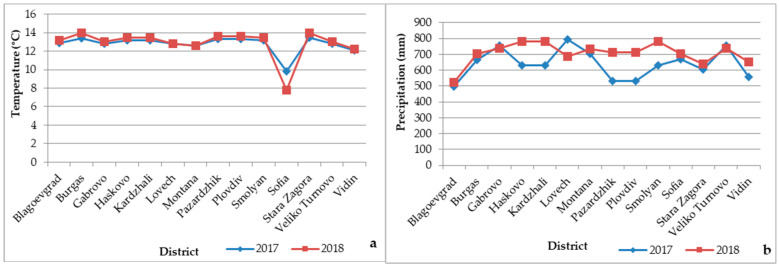
Variations in mean annual temperature (**a**) and precipitation (**b**) provided by Bulgarian meteorological district stations in 2017 and 2018.

In our study, snails were surveyed at altitudes ranging from 78 to 1926 m above sea level. For the 14 study districts, mean annual temperature and precipitation for 2017 and 2018 are presented in Figure 3a,b. The temperature range was from 7.8 °C to 14.0 °C, with close values for the two years studied *(p* > 0.05), with the lowest mean annual temperatures recorded in Sofia district. The range of precipitation was from 523 mm to 796 mm, with significant variation observed in half of the studied districts (Haskovo, Kardzhali, Lovech, Pazardzhik, Plovdiv, Smolyan and Vidin), during the two years of study (*p* < 0.05).

#### 3.2.2. Identification of Factors Related to the Presence of *G. truncatula* Snails

The logistic regression model identified temperature, shade and type of water body as factors related to the occurrence of *G. truncatula* (Table 4). *G. truncatula* snails were most likely to be found in sites located in streams and on the banks of small rivers, falling in temperature range of 12.2–12.8 °C. Snails were less likely to be found at sites completely shaded by trees and bushes. The best selected model explaining 12.4% of the variation. Odds ratios values, confidence intervals and *p*-values of the predictors are presented in Table 5.

## 4. Discussion

This study presents the results of the first large-scale targeted survey of *G. truncatula* occurrence in Bulgaria and Southeastern Europe. Previous records about the presence of *G. truncatula* on the Balkan Peninsula (summarized in Table 6) originate from small-scale surveys primarily focused on general gastropod faunal diversity in limited geographical areas, rather than targeted searches for *G. truncatula*. However, this snail species is common in this part of Europe, being found under different conditions across diverse types of freshwater bodies with a frequency ranging from 6.3% to 100.0%.

Our survey identified *G. truncatula* habitats in 56 of 191 sites (29.3%), confirming the species’ widespread distribution in Bulgaria [42]. The snail was recorded in three previously unreported regions: the Kazanlak Valley, Chepan Mountain, and Slavyanka Mountain, located within the districts of Stara Zagora, Sofia and Blagoevgrad, respectively. The spatial presence of *G. truncatula* did not differ significantly between the 14 studied districts of Bulgaria, indicating that the distribution of the snails was not influenced by the specific ecological features of the regions.

The global distribution of *G. truncatula* is facilitated by its high adaptability to a wide range of environmental conditions, which vary widely through the distribution range. In general, the relationships between altitude, temperature and precipitation are determined by different physical processes [43]. We found *G. truncatula* in habitats ranging from 78 to 1926 m above sea level. Furthermore, regression analysis indicates altitude as a parameter that is not related to the occurrence of *G. truncatula* in studied areas. Our finding is consistent with published data from regions in Transcarpathia [44] and Switzerland in Central Europe [45]. At the same time, Dreyfuss et al. [46] found a significant decrease in the frequency of snail populations with increasing altitude in regions in Central France (Western Europe). In the review by Smith et al. [18], altitude was identified as a factor with a mixed effect on the distribution of this snail species. *G. truncatula* is known to be present in ecosystems in South America up to 4100 m, which points to its great adaptability to local conditions [47].

Precipitation is considered an environmental parameter influencing the occurrence of *G. truncatula* [46]. During the period of our study, precipitation levels varied in half of the studied areas. A significant moderate negative correlation was found between precipitation and altitude. However, precipitation did not significantly affect snail occurrence. This finding aligns with the review by Smith et al. [18], where the impact of precipitation on the presence of *G. truncatula* is stated as controversial. This conclusion is supported by the mixed results showing a lack of association between these two parameters observed in Sweden [48] and Switzerland [45], a positive correlation described in Belgium [49] and a negative correlation found in France [46]. Snails are known to be significantly resistant to transient adverse conditions [50]. *G. truncatula* is a good aestivator and can survive in drought conditions [51]. High precipitation levels could increase water flow, which could damage snail habitats [52]. Precipitation plays a crucial role in geographical areas with dry climate, such as North Africa [53] or the Bolivian Altiplano [47], with its influence being related to the size of the habitat or the migration of snails, but not to the presence of the snails themselves [54].

Temperature is considered a limiting factor for the distribution of *G. truncatula*, with values between 10 and 25 °C being optimal for its development, although the exact optimal range remains uncertain [18]. During the period of our study, temperature values showed small deviations and were only weakly correlated with altitude. Data analysis identified temperature as an important factor associated with the occurrence of *G. truncatula,* with optimal values ranging between 12.2 °C and 12.8 °C. Similarly, in Central France, a significant increase in snail prevalence was observed with an increase in temperature from 9.5 °C to over 11 °C [46]. Also, in South Africa, *G. truncatula* is most widely distributed in cooler areas with temperatures ranging from 5 °C to 15 °C [55]. Collectively, these findings from different geographical regions indicate a preference of the species for lower temperatures, optimally between 10 °C and 15 °C. Temperature influences the development of snail egg masses as well as the growth of food sources [56]. It may also indirectly affect other microclimatic factors within or surrounding the habitats. In predictive models developed for the territory of Switzerland, all temperature-related variables show a negative effect on the occurrence of *G. truncatula* [45].

Field studies on the distribution of *G. truncatula* have typically focused on open water bodies, lacking shade from nearby trees or shrubs. The effect of shade on snail presence has rarely been investigated. Sunlight plays an important role in the growth of plants and algae, that serve as food sources for *G. truncatula* [57]. Our results indicate a pronounced preference for habitats with no or with partial shade. In Switzerland, the proximity of individual trees, shrubs or nearby forests has been identified as a factor positively associated with the local presence or absence of *G. truncatula* [45]. Our data also demonstrate the presence of snails in completely shaded sites, suggesting an ability to utilize algae and plants that grow under such conditions. Shade likely influences the microclimate, affecting snail activity and providing protection from desiccation, heat, temperature fluctuations, and ultraviolet radiation. However, habitats completely shaded by trees are significantly less likely to contain detectable eDNA [58] and egg masses [59] of *G. truncatula*. Based on the cited findings from Wales, Switzerland and Bulgaria, shade can therefore be considered is an important factor influencing the occurrence of *G. truncatula*. This conclusion is indirectly supported by local livestock management practices of moving herds from open fields, where trees or shrubs are scarce, to high mountain pastures, in order to reduce the risk of the animals being infected with “flukes”.

*G. truncatula* is well known for its ability to colonize a wide range of water bodies, including some very unusual habitats [28]. A total of 68 different habitat types have been described, with 20 identified as primary [18]. Our data show that *G. truncatula* prefers streams and banks of small rivers, which is consistent with previous reports [30,31,32,33,34,35,36,38,39,55]. However, this contradicts the generally accepted view that *G. truncatula* prefers muddy surfaces, also noted by Smith et al. [18]. According to these authors, this may be a consequence of an adaptation to new food other than blue-green algae growing on muddy surfaces.

In our study, artificial fountains used for watering domestic animals were the most frequently surveyed water bodies. Although *G. truncatula* occurred less often in fountains than in streams and small rivers, its presence in fountains can be described as common. Data on the presence of this species in fountains have also been reported for several Greek islands [32,33,34]. It is suggested that *G. truncatula* or its eggs are introduced into fountains by attaching to the hooves of animals traveling from nearby natural water sources or via bird transport [60]. Habitat features such as the constancy of clean, fresh water, water level, uniform flowing rate, bottom structure, plant/algae presence, etc., make fountains attractive for colonization by *G. truncatula*. In Switzerland, spring water has been identified as a significant predictor of *G. truncatula* presence [45]. Therefore, the occurrence of snails in these hotspots where domestic or wild ruminants gather increases the local risk of trematode infections. Overall, the available data indicate that habitat type is an important factor associated with snail occurrence [55].

## 5. Conclusions

This study mapped the spatial distribution of *G. truncatula* across different regions of Bulgaria and analyzed its relationship with various environmental factors such as altitude, temperature, precipitation, shading and water body type. The results identify temperature, shade and type of water body as key limiting factors influencing snail distribution. These findings help clarify the species’ habitat preferences, which appear to be relatively consistent. Consequently, this study provides new insights into the ecological flexibility and potential for global spread of *G. truncatula*.

*G. truncatula* demonstrated remarkable adaptive potential, allowing it to colonize atypical habitats with a wide range of environmental conditions. However, understanding this adaptability requires the analysis of both linear and nonlinear models. Our data fill a significant knowledge gap for a large biogeographic region and can support the assessment of current risks of trematode infections as well as the prediction of future outbreaks. This represents an important step forward in controlling major parasitic diseases such as fasciolosis and paramphistomosis in ruminants across Bulgaria and Southeastern Europe.

## Figures and Tables

**Table 1:** Number of surveyed water points in July–August 2017 and July–August 2018 according to district and number of positive sites for *G. truncatula* (in parentheses).

District	Number of Sites Surveyed (with Snails)	Frequency (%)
Blagoevgrad	44 (15)	34.1
Burgas	2 (1)	50.0
Gabrovo	10 (5)	50.0
Haskovo	13 (2)	15.4
Kardzhali	21 (4)	19.0
Lovech	9 (3)	33.3
Montana	9 (2)	22.2
Pazardzhik	5 (1)	20.0
Plovdiv	6 (2)	33.3
Smolyan	7 (1)	14.3
Sofia	37 (11)	29.7
Stara Zagora	5 (2)	40.0
Veliko Turnovo	21 (6)	28.6
Vidin	2 (1)	50.0
Total	191 (56)	29.3

**Table 2:** Values of Pearson’s correlation coefficients for altitude, temperature and precipitation in the 14 study districts. CI—Confidence Intervals.

Parameters Pearson’s Coefficients (95% CI)	Altitude	Mean Annual Temperature
Mean annual temperature	−0.202 (−0.335; −0.062) *	-
Mean annual precipitation	−0.402 (−0.514; −0.276) **	−0.001(−0.143; 0.140)

* *p* < 0.01; ** *p* < 0.001.

**Table 3:** Number and frequency (%) of *G. truncatula* habitats in selected categories of altitude, mean annual temperature, mean annual precipitation, presence of shade and type of water body in 14 districts of Bulgaria in 2017 and 2018.

Parameter	Altitude Range (m.a.s.l.)			Temperature Range (°C)			Precipitation Range (mm)			Shade			Type of Water Body					Total
	< 500	501– 1000	> 1000	7.8, 9.8	12.2–12.8	13.0–14.0	523, 532	603– 686	702– 796	No	Partial	Yes	^p^ A	^p^ B	^p^ C	^p^ D	^p,t^ E	
Number of sites with *G. truncatula*	26	25	5	11	17	28	16	13	27	36	17	3	28	5	12	6	5	56
Number of sites without *G. truncatula*	68	55	12	26	28	81	34	29	72	84	28	23	88	24	8	6	9	135
Total	94	80	17	37	45	109	50	42	99	120	45	26	116	29	20	12	14	191
Frequency (%)	27.7	31.3	29.4	29.7	37.8	25.7	32.0	31.0	27.3	30.0	35.6	11.5	24.1 *	17.2 *	60.0 *	50.0	35.7	29.3

m.a.s.l.—Meters above sea level; A, B—Fountains for animal watering; C—Streams; D—Small rivers; E—Other (^p^ Spring heads (a total of 5 (2)), ^p^ Irrigation ditches (a total of 7 (1)) and ^t^ puddles (a total of 2 (2)); ^p^—perennial water body, ^t^—temporary water body; In parentheses is the number of positive sites for *G. truncatula*. * Significant difference between type A and type C, and between type B and type C (*p* < 0.01).

**Table 4:** Output of the logistic regression to model the occurrence of *G. truncatula.*

Variables	Estimate	Standard Error	Z Value	Pr (>|z|)
Intercept	−2.85	1.206	−2.363	0.018 *
Altitude 501–1000 m	0.624	0.536	1.163	0.245
Altitude > 1000 m	0.902	0.71	1.27	0.2
Temperature 12.2–12.8 °C	2.385	0.983	2.427	0.015 *
Temperature 13.0–14.0 °C	1.2888	0.887	1.453	0.146
Precipitation 603–686 mm	0.632	0.826	0.766	0.444
Precipitation 702–796 mm	−0.139	0.523	−0.265	0.791
Shade Partial	0.269	0.428	0.643	0.52
Shade Yes	−1.615	0.727	−2.222	0.026 *
Water_body Fountains type B	−0.29	0.562	−0.516	0.606
Water_body Streams	1.62	0.559	2.895	0.004 **
Water_body Small rivers	1.638	0.735	2.227	0.026 *
Water_body Other	0.612	0.648	0.943	0.346

Significance codes: * 0.05; ** 0.01.

**Table 5:** Odd ratios, confidence intervals and *p*-values for predictors influencing the outcome, as determined by logistic regression analysis.

Predictors	Odds Ratios	Confidence Intervals	*p*-Value
Temperature 12.2–12.8 °C	3.12	1.04–10.12	0.048 *
Temperature 13.0–14.0 °C	1.57	0.59–4.53	0.381
Shade Partial	1.43	0.64–3.13	0.378
Shade Yes	0.26	0.05–0.90	0.054
Water_body Fountains type B	0.66	0.20–1.84	0.458
Water_body Streams	4.85	1.73–14.57	0.003 **
Water_body Small rivers	4.64	1.14–19.52	0.032 *
Water_body Other	2.13	0.59–7.12	0.227
Observations = 191 AIC = 224.920 R^2^ Tjur = 0.124			

* *p* < 0.05; ** *p* < 0.01.

**Table 6:** Number and type of water bodies surveyed and frequency (%) of *G. truncatula* habitats in studies from the Balkan countries.

Country	Number and Type (Superscript) of Water Bodies Studied	Frequency (%)	References
Bulgaria	191 ^1,2,3,4,7^	29.3	This study
Bosnia and Herzegovina	23 ^2,5,7^	8.0	[28]
	19 ^2^	52.6	[29]
Croatia	14 ^4^	7.1	[30,31]
	49 ^2,3,4,7^	71.4	
Greece	35 ^1,2,3,5,7^	28.6	[32]
	6 ^1,2,4^	16.7	[33]
	27 ^1,2,3,7^	14.8	[34]
Romania	17 ^2,3,7^	35.3	[35]
	16 ^4,6^	6.3	[36]
	5 ^5^	20.0	[37]
	4 ^2,4^	20.0	[38]
Serbia	15 ^4^	20.0	[39]
Turkey	5 ^5^	80.0	[40,41]
	5 ^5^	100.0	

^1^—Fountains; ^2^—Springs; ^3^—Streams/Rivulets; ^4^—Rivers/Brooks/Small rivers; ^5^—Lakes; ^6^—Swampy areas; ^7^—Others.

## Data Availability

The original contributions presented in this study are included in the article/Supplementary Materials. Further inquiries can be directed to the corresponding author.

## Supplementary Materials

The following supporting information can be downloaded at: https://www.mdpi.com/article/10.3390/ani16020226/s1. Table S1: Dataset of sampling sites, date, GPS coordinates, altitude, mean temperature, mean precipitation, shade cover, type of water body and presence of *Galba truncatula*.
